# Physicochemical Properties and Biocompatibility of Electrospun Polycaprolactone/Gelatin Nanofibers

**DOI:** 10.3390/ijerph18094764

**Published:** 2021-04-29

**Authors:** Wei Lee Lim, Shiplu Roy Chowdhury, Min Hwei Ng, Jia Xian Law

**Affiliations:** Centre for Tissue Engineering and Regenerative Medicine, Faculty of Medicine, Universiti Kebangsaan Malaysia Medical Centre, Jalan Yaacob Latif, Kuala Lumpur 56000, Malaysia; sabrinalim36@gmail.com (W.L.L.); shiplu56@gmail.com (S.R.C.); angela@ppukm.ukm.edu.my (M.H.N.)

**Keywords:** polycaprolactone, gelatin, tendon, ligament, tissue engineering, electrospinning, aligned nanofibers

## Abstract

Tissue-engineered substitutes have shown great promise as a potential replacement for current tissue grafts to treat tendon/ligament injury. Herein, we have fabricated aligned polycaprolactone (PCL) and gelatin (GT) nanofibers and further evaluated their physicochemical properties and biocompatibility. PCL and GT were mixed at a ratio of 100:0, 70:30, 50:50, 30:70, 0:100, and electrospun to generate aligned nanofibers. The PCL/GT nanofibers were assessed to determine the diameter, alignment, water contact angle, degradation, and surface chemical analysis. The effects on cells were evaluated through Wharton’s jelly-derived mesenchymal stem cell (WJ-MSC) viability, alignment and tenogenic differentiation. The PCL/GT nanofibers were aligned and had a mean fiber diameter within 200–800 nm. Increasing the GT concentration reduced the water contact angle of the nanofibers. GT nanofibers alone degraded fastest, observed only within 2 days. Chemical composition analysis confirmed the presence of PCL and GT in the nanofibers. The WJ-MSCs were aligned and remained viable after 7 days with the PCL/GT nanofibers. Additionally, the PCL/GT nanofibers supported tenogenic differentiation of WJ-MSCs. The fabricated PCL/GT nanofibers have a diameter that closely resembles the native tissue’s collagen fibrils and have good biocompatibility. Thus, our study demonstrated the suitability of PCL/GT nanofibers for tendon/ligament tissue engineering applications.

## 1. Introduction

The Global Burden of Disease Study in 2016 has reported approximately 1.27 billion cases of musculoskeletal disorders, with years living with disability to be around 137 million [1]. Of these, tendon and ligament injury, especially from sport-related injuries, is prevalent and poses a massive burden on society and the economy. More than 300,000 patients underwent surgery for an injured tendon or ligament in the United States alone in 2013 [2]. Surgical intervention for repairing damaged tendons or ligaments varies, from simple ligation to requiring tissue graft from various sources (autograft, allograft, xenograft) [3]. However, complications related to the graft have limited its usage. Autograft is associated with donor site morbidity, while allograft and xenograft have the risk of pathogen transmission and immunogenic reaction. A synthetic graft is associated with early rupture with insufficient tissue ingrowth [4,5]. Thus, there is a need to find a suitable alternative to compensate for the current graft drawbacks.

In recent years, the applications of tissue engineering products for tendon or ligament replacement are not uncommon. Numerous engineered tissues have been fabricated from various biomaterials, both natural and synthetic such as silk, alginate, chitosan, polycaprolactone (PCL), polyglycolic acid, and polylactic acid to mimic the native tendon or ligament structure [6,7,8]. Such an example includes the work of Petrigliano et al., who successfully electrospun PCL for ligament reconstruction in rodents [9]. PCL is a synthetic polymer known for its durability in various biomedical applications such as sutures, prosthetics, and even approved by the FDA for human use [10,11]. It has appropriate mechanical strength in bulk [12], good processability [11], highly modifiable [13], and biodegradable [13]. PCL is biocompatible and has mild undesirable host reactions [13]. However, PCL has poor cellular attachment and proliferation owing to its hydrophobicity, inadequate wettability, and lack of bioactive functional groups [14,15].

Several methods such as the use of nanofillers, surface modifications and polymer blends were introduced to complement PCL’s shortfall [16,17,18]. As such, many have implemented the idea of combining the durable synthetic polymer with a natural polymer that is cell-friendly. Natural polymers have characteristics more closely resembling the native extracellular matrix (ECM). Natural polymers provide various bioactive functional groups to accommodate cell attachment, proliferation, and infiltration. Xu et al. used an electrospinning technique to prepare a PCL/collagen scaffold that showed promising results, portrayed by the scaffold’s good porosity, sufficient mechanical strength, and increased tenocyte infiltration, proliferation, and ECM gene expression [19].

In this study, we attempted to fabricate PCL with gelatin (GT) for tendon and ligament replacement. GT is a natural polymer produced by collagen’s partial hydrolysis and can be sourced from animals and plants. It shares many similar properties as its predecessor. Most importantly, GT is less immunogenic than collagen, attributed to its lack of specific amino acids such as tyrosine, tryptophan, and phenylalanine that mediate immunogenic reactions [20].

Tendon and ligament ECM are composed of dense and aligned collagen fibrils. The ECM’s unique structural orientation has been proven to direct the cell alignment, which contributes to the tissue’s functionality. The alignment is believed to provide topographical cues for the cell to perform its functions, namely attachment, proliferation, and differentiation [21,22]. Electrospinning is a relatively simple technique that produced aligned nanofibers mimicking the tendon and ligament tissue’s aligned structural matrix. The electrospinning process involves applying biomaterial solution to a strong electric field through a narrow nozzle that can generate ultra-fine fiber ranging from a few micrometers to tens of nanometers in diameter [23]. Therefore, in this study, blended PCL and GT were electrospun to create a suitable scaffold that could serve as a potential tissue replacement for tendons and ligaments.

## 2. Materials and Methods

### 2.1. Fabrication of PCL/GT Nanofibers

**Figure 1:**
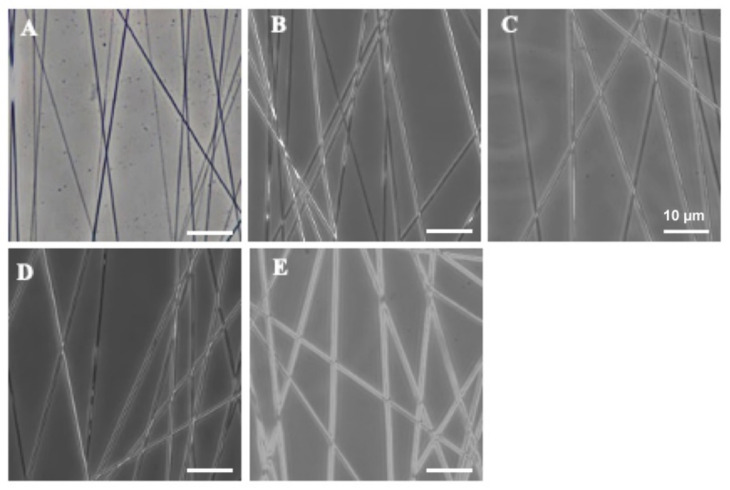
Phase-contrast images showing fabricated PCL/GT nanofibers at different ratio (**A**) 100:0, (**B**) 70:30, (**C**) 50:50, (**D**) 30:70, (**E**) 0:100.

The electrospinning technique was used to prepare nanofibers from a blended mixture of PCL (Sigma, USA) and GT (Nitta Gelatin, Japan). Briefly, PCL was dissolved in 2,2,2-trifluoroethanol (TFE; Sigma) to prepare 6% (*w*/*v*) solution. The solution was magnetically stirred for 24 h until the polymers dissolve completely. GT was dissolved using TFE to prepare a 10% (*w*/*v*) solution. A mixture of the solution was then prepared at 100:0, 70:30, 50:50, 30:70, and 0:100 ratios. The solution mixture was electrospun at an applied voltage of 5 kV, a flow rate of 0.05 mL/h, 45 min of spinning duration, and 20 cm needle-to-collector distance onto a 1000 rpm rotating collector (4.6 × 8 × 4.6 cm) that yielded aligned nanofibers without beadings (Figure 1). The resulting PCL/GT nanofibers were air-dried under a biosafety cabinet to allow for complete solvent evaporation, UV-irradiated, and soaked with 1% antibiotic-antimycotic solution followed by incubation in complete culture medium prior to any experimental use.

### 2.2. Nanofiber Structural Analysis

Scanning electron microscopy (SEM) (VPSEM LEO 1450, Carl Zeiss, Oberkochen, Germany) was performed to study the alignment and diameter of the fiber. Briefly, samples were fixed in 2.5% glutaraldehyde solution to preserve their biological structure, dehydrated with graded acetone series, critical point dried, and sputter-coated with conductive metal before visualization using SEM. Fiber diameter was determined by measuring the diameter of the captured SEM images using ImageJ software (National Institutes of Health, Bethesda, MD, USA) [24]. At least 50 fibers were analyzed per sample. The fiber alignment of electrospun PCL/GT fibers was determined by measuring the fibers’ angle relative to the horizontal axis on SEM micrographs. For each sample, the angles of a minimum of 70 individual fibers were measured using NIS-Elements software (Nikon, Tokyo, Japan). Fiber alignment angles were then normalized to 0° (horizontal axis) and plotted as histograms. The normalized angle’s positive and negative values indicate the opposite orientation of fibers relative to the horizontal axis. 

### 2.3. Post-Immersion Morphological Investigations of PCL/GT Nanofibers

The PCL/GT nanofibers were submerged in phosphate-buffered saline (PBS; Sigma) of pH 7.4, and images were taken using the Nikon A1 confocal microscope (Nikon, Tokyo, Japan) on days 0, 2, 4, and 6. At least five images were taken from each well and analyzed for fiber disintegration. 

### 2.4. Contact Angle Measurement

The contact angle was measured using the modified version of the sessile drop method to determine the hydrophilic/hydrophobic properties of the PCL/GT nanofibers [25]. Briefly, the nanofibers were laid on a flat surface. A drop of distilled water was dropped onto the surface, and a picture of the water contact to the surface was captured. Images were analyzed for the contact angle of the water droplet using ImageJ software.

### 2.5. Fourier Transform Infrared Spectroscopy (FTIR)

FTIR analysis of the nanofibers was performed to characterize the functional groups in the nanofibers. FTIR data were recorded on a Nicolet Nexus 470 FTIR spectrometer (Thermo Fisher Scientific, Waltham, MA, USA). The FTIR spectrometer was purged continuously with nitrogen. A total of 64 scans were collected with a resolution of 2 cm^−1^. The infrared spectra were recorded in transmission mode using a PCL/GT nanofiber deposited on a silicon wafer.

### 2.6. Energy Dispersive X-ray Spectroscopy (EDX)

EDX analysis (Oxford Instruments, Abingdon, UK) was performed to detect the percentage of every element on the nanofibers. This is based on the principle that every chemical element possesses a unique atomic structure distinguished by a unique electromagnetic emission upon excitation with an X-ray.

### 2.7. Wharton’s Jelly-Derived Mesenchymal Stem Cell (WJ-MSC) Isolation and Culture

Umbilical cord samples were collected from six healthy donors with a full-term pregnancy of 38-40 weeks and delivered by elective cesarean section in the Universiti Kebangsaan Malaysia Medical Centre (PPUKM). This study’s ethical approval was obtained from the Research Ethics Committee of Universiti Kebangsaan Malaysia (approval project code: UKM PPI/111/8/JEP-2017-396). Cell isolation and culture were performed as described previously [26,27]. Briefly, the umbilical cord was cleaned with a sterile scalpel to remove both umbilical arteries and the vein. Wharton’s jelly was then minced into 2 mm^2^ pieces and digested with 0.6% (*w*/*v*) collagenase type II (Worthington, Lakewood, NJ, USA) in an incubator shaker (250 rpm/h) at 37 °C for 1 h. The digested tissue was then centrifuged, and the pelleted cells were re-suspended in low glucose DMEM (Invitrogen, Carlsbad, CA, USA) supplemented with 10% fetal bovine serum (FBS) and cultured in the 6-well plate. All cultures were maintained in a humidified incubator at 5% CO_2_ and 37 °C. The culture medium was changed every 3 days.

### 2.8. Cell Viability

The viability of the WJ-MSCs cultured on the PCL/GT nanofibers at different culture periods (days 1, 4, and 9) was determined using a resazurin-based viability assay kit (Sigma-Aldrich, St. Louis, MI, USA). Briefly, WJ-MSCs were seeded on the PCL/GT nanofibers at a density of 3000 cells/cm^2^. Cell-seeded PCL/GT nanofibers were incubated in the dark for 3 h in the Resazurin dye solution. The resulting supernatant from each sample was transferred into a 96-well plate and quantified with a spectrophotometer. The absorbance readings at 600 nm were recorded.

### 2.9. Cell Morphology and Alignment

Images of the WJ-MSCs cultured on the nanofibers were captured using the Nikon A1 confocal microscope. For each sample, a minimum of 60 cells were measured for their angles using NIS-Elements software. Cell alignment angles were then normalized to 0° (horizontal axis) and plotted as histograms. The normalized angle’s positive and negative values indicate the opposite orientation of cells relative to the horizontal axis.

### 2.10. Tenogenic Expression by Quantitative Polymerase Chain Reaction (PCR)

In this experiment, WJ-MSCs were cultured in three different groups. The first group was the control group, whereby WJ-MSCs were cultured without PCL/GT nanofibers and tenogenic induction. The second group was the culture of WJ-MSCs on PCL/GT 70:30 nanofibers without tenogenic induction. The third group was the culture of WJ-MSCs on PCL/GT nanofibers with tenogenic induction. Briefly, for tenogenic induction, once the culture reached 80% confluency, it was starved for 12 h with DMEM supplemented with 1% fetal bovine serum (FBS) followed by treatment with 100 ng/mL recombinant BMP-12 in the same medium for 48 h. After 3 days of culture, total RNAs were extracted using the RNeasy Plus Mini kit (Qiagen, Hilden, Germany) following the manufacturer’s instructions. Isolated RNA was reverse transcribed with the QuantiNova Reverse Transcription kit (Qiagen), and the cDNA was subjected to real-time PCR with QuantiNova SYBR Green PCR kit (Qiagen, Hilden, Germany). The primers used for PCR are scleraxis (SCX) (NM_001080514-F: CTGGCCTCCAGCTACATCTC, R: CTGAGGCAGAAGGTGCAGAT); tenomodulin (TNMD) (NM_022144.3-F: CCCAGCAGAAAAGCCTATTG, R: GCGTGACGGGTCTTCTCTAC); tenascin-C (TNC) (NM_002160.4–F: TTCACTGGAGCTGACTGTGG, R: TAGGGCAGCTCATGTCACTG); decorin (DCR) (BT019800.1–F: AATTGAAAATGGGGCTTTCC, R: GCCATTGTCAACAGCAGAGA), glyceraldehyde 3-phosphate dehydrogenase (GAPDH) (BT006893.1-F: GAGTCAACGGATTTGGTCGTR: TTGATTTTGGAGGGATCTCG). GAPDH was used as the housekeeping gene.

### 2.11. Statistical Analysis

All data were presented as the mean ± standard error of the mean (SEM) of three biological replicates (*n* = 3). Statistical analysis was performed using GraphPad Prism (version 7.0). One-way and two-way analysis of variance (ANOVA) was used to compare the results of multiple groups with the post-hoc Tukey or Dunnett’s test. A *p*-value of less than 0.05 was considered significant.

## 3. Results

### 3.1. Physical Characterization of PCL/GT Nanofibers

**Figure 2:**
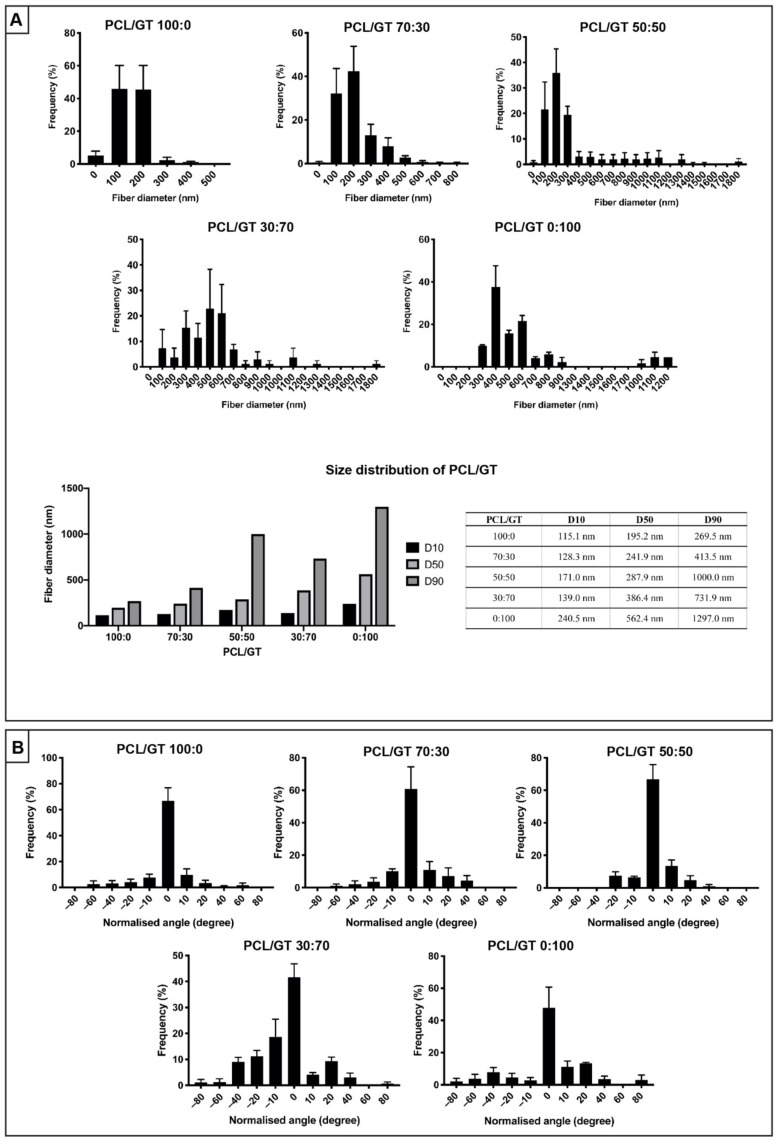
(**A**) The diameter and size distribution of PCL/GT nanofibers. Note that increasing GT concentration will increase the fiber diameter. (**B**) The fiber alignment of PCL/GT nanofibers. All nanofibers were relatively aligned, as indicated at the 0° normalized angle.

Electrospun PCL/GT nanofibers at different ratios yielded various fiber diameter distributions (Figure 2A). Only PCL/GT 100:0, 70:30, and 50:50 have most nanofibers within the diameter range of 100–200 nm. As the gelatin ratio increases, the resulting nanofibers mean diameter also increases. For nanofiber alignment, all nanofibers at different ratios were relatively aligned at the pre-set horizontal axis (normalized degree) (Figure 2B). PCL/GT 100:0 and PCL/GT 50:50 showed the highest frequency of aligned nanofibers (66.77 ± 17.35%, 66.79 ± 15.53%) followed by PCL/GT 70:30 (60.71 ± 23.64%), PCL/GT 0:100 (47.76 ± 22.49%) and PCL/GT 30:70 (41.62 ± 9.00%).

**Figure 3:**
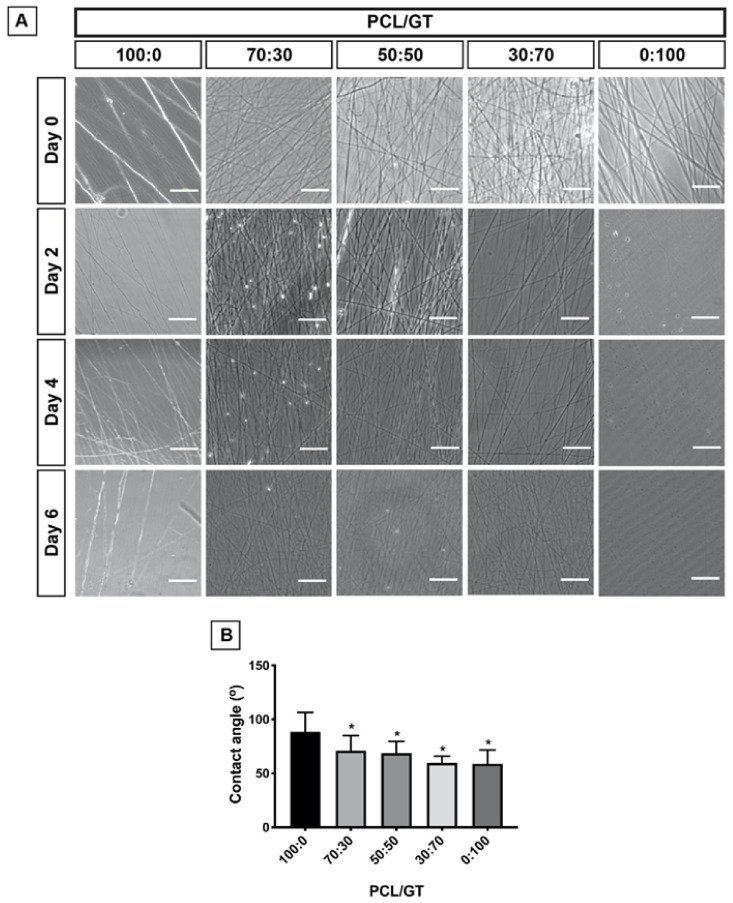
(**A**) Post-immersion morphological investigations of PCL/GT nanofibers in phosphate-buffered saline at different time points. (Scale bar represents 100 µm). (**B**) The water contact angle of PCL/GT nanofibers. One-way ANOVA with post-hoc Tukey was used for analysis. * *p*-value (<0.05).

Pure GT nanofibers (PCL/GT 0:100) were degraded entirely by day 2 (Figure 3A). Meanwhile, nanofibers containing PCL (PCL/GT 100:0, 70:30, 50:50, and 30:70) were not degraded up to day 6. However, microscopic images revealed nanofiber “wrinkling” at day 2 (PCL/GT 100:0), day 4 (PCL/GT 70:30 and PCL/GT 50:50), and day 6 (PCL/GT 30:70). 

The PCL/GT nanofibers were tested for their hydrophilicity. An angle of >90° indicates a tendency to hydrophobicity, while an angle of < 90° indicates hydrophilicity. All PCL/GT nanofibers have an angular value below < 90°, whereby PCL/GT 100:0 showed the highest angular value (88.5 ± 17.9°) approaching hydrophobicity (Figure 3B). Statistical analysis showed that PCL/GT 70:30 (71.0 ± 14.0°), PCL/GT 50:50 (68.7 ± 10.9°), PCL/GT 30:70 (59.6 ± 6.3°) and PCL/GT 0:100 (58.9 ± 12.8°) were significantly more hydrophilic than PCL/GT 100:0 (*p* < 0.05). Post-hoc analysis revealed that increasing GT concentration did not significantly increase hydrophilicity.

### 3.2. Chemical Characterization of PCL/GT Nanofibers

**Figure 4:**
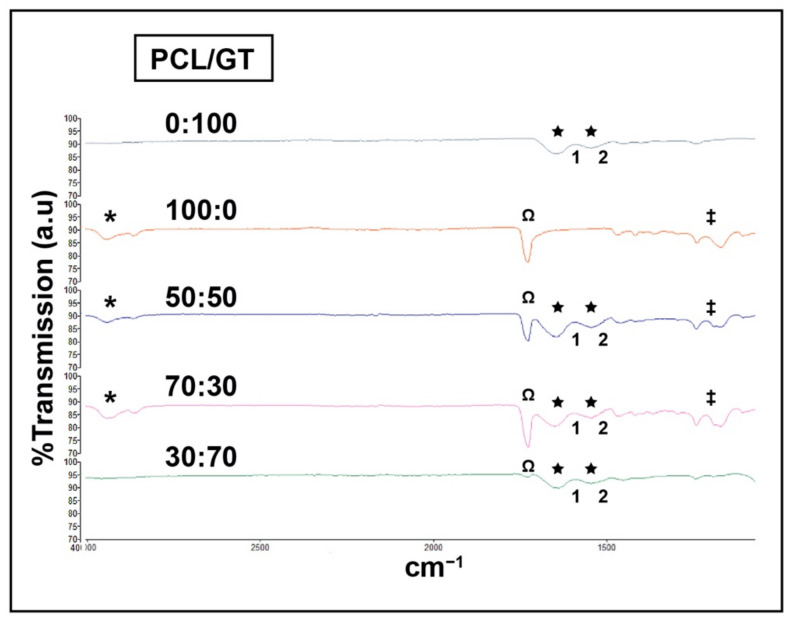
FTIR spectra of PCL/GT nanofibers. Blended composite PCL and GT contained both polymer’s spectrum as indicated by the distinctive peaks. * H-C-H stretching, Ω carbonyl (C=O) stretching, ‡ C-O-C stretching indicates PCL presence. ★ Common bands of protein (indicates the presence of GT); **1**. C-O stretching, **2**. N-H and C-N (peptide bond) stretching.

Figure 4 demonstrates the functional groups found in the composite nanofibers to confirm PCL and GT’s presence through FTIR spectra. At least two distinct peaks were observed at 1653 and 1552 cm^−1^ in pure gelatin (PCL/GT 0:100) that represent the common bands of protein. Four absorption peaks at 2940 (H-C-H stretching), 1722 (carbonyl (C=O) stretching), 1293 and 1240 (C-O-C stretching) cm^−1^ were detected in pure PCL nanofibers (PCL/GT 100:0). Regarding the composite PCL/GT nanofibers (70:30, 50:50, 30:70), all six absorption peaks were found in the FTIR spectra, indicating the presence of both PCL and GT.

The EDX test was conducted to determine the PCL/GT nanofibers’ elemental percentage. Meta-comparison of the carbon composition revealed a significant drop in carbon percentage in the pure GT nanofibers (PCL/GT 0:100) as compared to the other groups (*p* < 0.05) (Table 1). A significant increase in the nitrogen element in pure GT nanofibers was observed compared to PCL/GT 100:0 and PCL/GT 70:30 (*p* < 0.05). No significant changes were detected in oxygen percentage. 

### 3.3. The Effect of PCL/GT Nanofibers on WJ-MSCs

**Figure 5:**
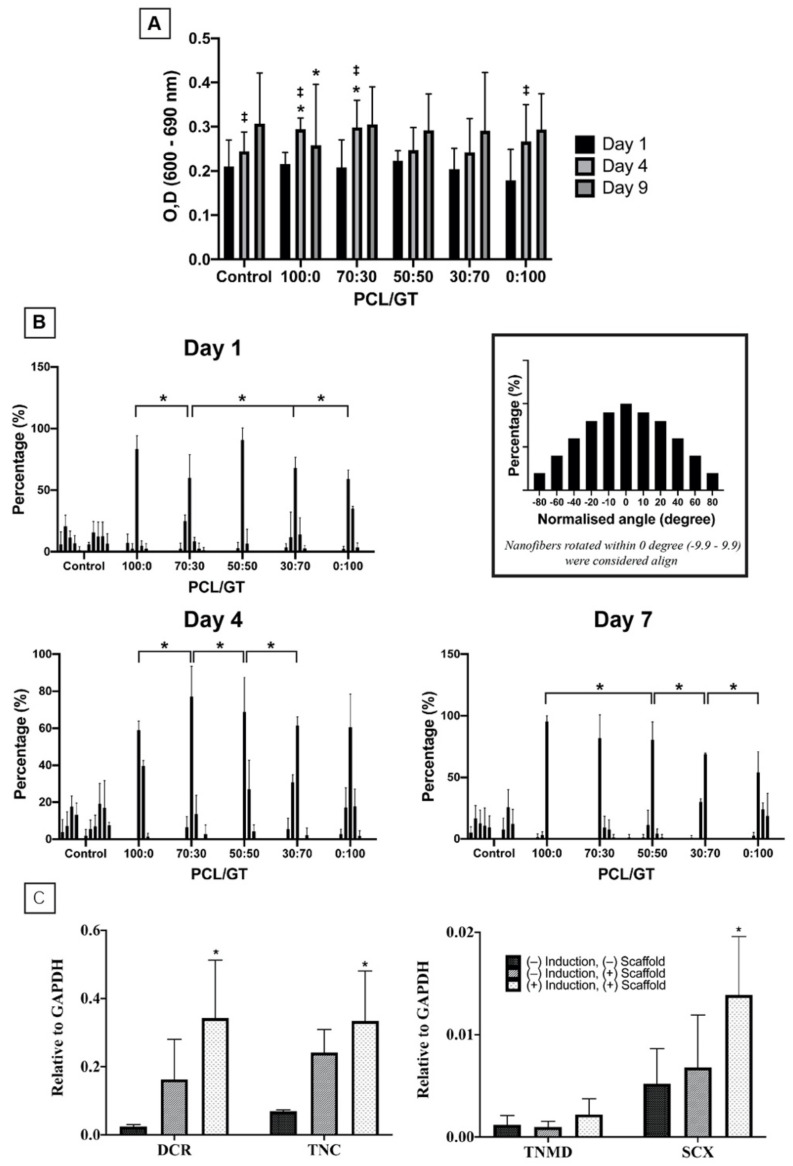
(**A**) Viability of WJ-MSCs cultured on PCL/GT nanofibers. Overall, WJ-MSCs were viable for 7 days in culture with PCL/GT nanofibers. Two-way ANOVA with post-hoc Tukey or Dunnett’s test was used for analysis. ‡ *p*-value (<0.05) compared to day 1 within each group. * *p*-value (<0.05) compared to control within each day (1, 4 and 9). (**B**) Alignment of WJ-MSCs on PCL/GT nanofibers. * *p*-value (<0.05). (**C**) Tenogenic gene expression of WJ-MSC in culture with PCL/GT nanofibers that includes decorin (DCR), tenascin-C (TNC), scleraxis (SCX) and tenomodulin (TNMD).

WJ-MSCs cultured on top of the PCL/GT nanofibers remained viable for up to 7 days. The viable cells in PCL/GT nanofibers were similar in count on day 1 compared to control. On day 4, PCL/GT 100:0 and 70:30 showed significant increase in viable cells compared to control (*p* < 0.05). On day 9, there was a significant decrease of viable cells in PCL/GT 100:0 in comparison to the control (*p* < 0.05), however, no significant drop in viability was seen in comparison to day 6 within the same group. Within control, PCL/GT 100:0, PCL/GT 70:30 and PCL/GT 0:100 groups, there were significant increase of cells in day 4 compared to day 1 (*p* < 0.05) (Figure 5A).

**Figure 6:**
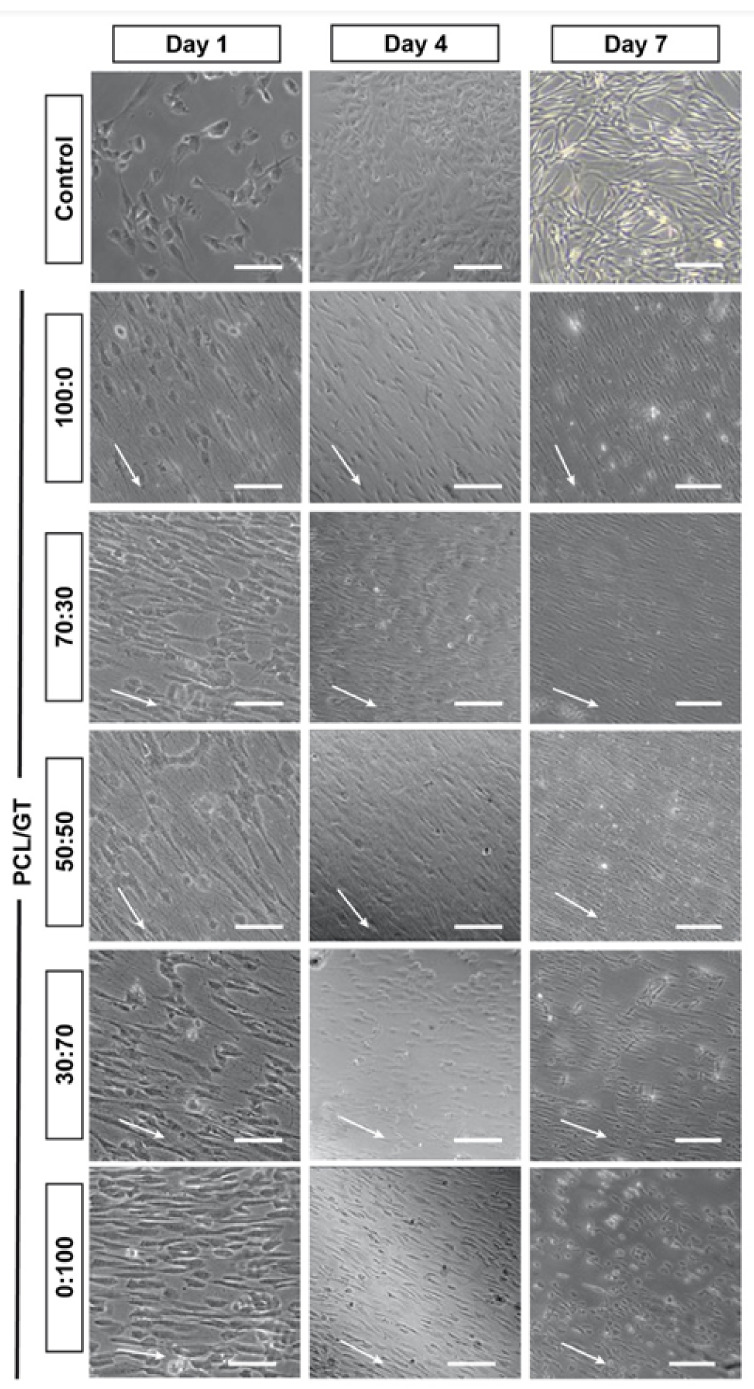
The alignment of WJ-MSCs on PCL/GT nanofibers. Note that WJ-MSCs on PCL/GT 0:100 started to lose their orientation as the nanofibers were degraded following days in culture. (Scale bar represents 100 µm, arrow indicates the direction of cell alignment).

The topographical effects of PCL/GT nanofibers on WJ-MSC alignment were studied by culturing the WJ-MSCs on top of the nanofibers. As illustrated in Figure 5B, only WJ-MSCs cultured without PCL/GT nanofibers resulted in random cell orientation, in contrast to the other groups that demonstrated aligned cell orientation on days 1, 4, and 7 (Figure 6). On day 1, PCL/GT 70:30 has a significantly lower percentage of aligned cells compared to PCL/GT 100:0, PCL/GT 30:70 and PCL/GT 0:100 (*p* < 0.05). On day 4, PCL/GT 70:30 has significant higher percentages of aligned cells compared to PCL/GT 100:0, while PCL/GT 50:50 < PCL/GT 70:30 and PCL/GT 30:70 < PCL/GT 50:50 (*p* < 0.05). On day 7, a similar observation was seen where PCL/GT 50:50 < PCL/GT 100:0, PCL/GT 30:70 < PCL/GT 50:50, and PCL/GT 0:100 < PCL/GT 30:70 (*p* < 0.05), indicating that increasing the GT ratio would decrease the percentage of aligned cells. 

The effects of PCL/GT nanofibers on the expression of tenogenic genes were also investigated. The genes studied were DCR, TNC, TNMD, and SCX which are the most common genes expressed in tenocytes. In this study, the nanofibers alone do not elicit a significant increase in DCR, TNC, TNMD, and SCX (*p* > 0.05) (Figure 5C). However, together with tenogenic induction, there was a significant increase in DCR, TNC, and SCX compared to the control group (*p* < 0.05). These results showed that the nanofibers supported the tenogenic differentiation of WJ-MSCs.

## 4. Discussion

The present study has demonstrated PCL/GT nanofiber characteristics for potential use in tendon/ligament tissue engineering, especially in terms of cellular attachment, alignment, and survivability. One of the fundamental components of tissue engineering is the scaffold that provides an appropriate and supportive microenvironment to regulate cell biology and neo-tissue formation. The scaffold should serve similar functions as the ECM, which acts as the home for cells to proliferate, migrate, and differentiate.

Various processing techniques, including emulsification/freeze-drying, thermally-induced phase separation, gas foaming, melt mixing and particle leaching, and electrospinning, have been implemented to produce a PCL-based scaffold [28]. It is important to note that different techniques will produce different features, i.e., porous scaffold and aligned fibrous scaffold. Therefore, it is vital to choose the technique that is best suited to produce the type of scaffold that mimics the intended native tissue. In the last few decades, electrospinning technique has been recognized as an efficient processing method for several applications such as bioremediation (as potent adsorbent for pollutants) [29], and biomedical/tissue engineering (aligned fibers) [11] owing to its ability to manufacture nanoscale fibrous structures. At the beginning of the study, the blended composite of PCL and GT was successfully fabricated via electrospinning. Electrospinning was chosen because it could fabricate aligned fibers that resemble the collagen structural assembly in tendon and ligament. The parameter settings (applied voltage, polymer flow rate, and needle-to-collector distance) were first optimized to achieve the desired nanofibers with straight and smooth morphology and without bead defects. These settings are necessary as different values will affect the fiber characteristics, especially the fiber diameter [30].

Tendon/ligament consists of defined bands of collagen fibers constituted from networks of collagen fibrils, the basic unit of the tendon/ligament tissue [31]. These collagen fibrils’ diameter ranges from 40–400 nm as viewed under transmission electron microscopy [32]. The diameter of the PCL/GT nanofibers mostly fall within a range of 100–600 nm. An interesting observation is that the nanofiber diameter increased relatively as GT concentration increased. Previous studies have also reported similar observations [33,34]. Most of the PCL/GT nanofibers were aligned and further validated the electrospinning’s main advantage in producing aligned nanofibers. Fiber alignment is considered one of the crucial factors in developing a scaffold for tendon/ligament replacement, as aligned nanofiber positively affects the tensile properties and resists damage against shear force [35].

Previous investigations have reported the durability of PCL nanofibers in biodegradation [36,37]. PCL nanofibers were said to withstand up to 6 months in vitro and 90 days in vivo with minimal mechanical strength changes [38]. Meanwhile, another study observed a faster degradation rate of composite nanofibers in the presence of GT, similar to our observation [39]. In this study, the PCL/GT nanofibers were subjected to immersion in PBS for 6 days and the fibers’ morphological changes were observed as an indirect proof of degradation. The water contact angle test has shown that the PCL/GT nanofibers are hydrophilic, although PCL/GT 100:0 was almost reaching the hydrophobicity threshold. A significant drop in the contact angle was observed as the GT concentration increased compared to control, highlighting GT as a hydrophilic biopolymer. Fetching appropriate polymer hydrophilicity is very important as it has been found to influence cellular adhesion, proliferation, and differentiation [40].

Analysis of the nanofiber’s chemical composition was conducted to ensure the presence of the polymers and to discern any possible chemical modification or interaction during the fabrication process. Chemical composition was evaluated via two different parameters: functional groups through FTIR and chemical elements via EDX. The elemental composition found on the PCL/GT nanofibers confirmed the integration of PCL and GT by observing the increment of nitrogen percentage representing GT’s presence. On the other hand, FTIR analysis has shown PCL and GT’s distinctive peaks, as reported from previous literature [41,42,43]. The spectra showed major peaks for PCL, 1722 cm^−1^ (carbonyl (C=O) stretching), 1293 cm^−1^ (C-O and C-C stretching), 1240 cm^−1^ (asymmetric C-O-C stretching) and 2940 cm^−1^ (asymmetric CH2 stretching). The major peaks for gelatin at 1653 and 1552 cm^−1^ corresponding to the amide I and amide II, respectively. The pure PCL solution peaks demonstrated the crystalline nature of PCL, while the gelatin slope represents its amorphous structure [31]. Meanwhile, PCL/GT 70:30, 50:50, and 30:70 did not show similar peaks to those of pure PCL and GT. Instead, eight different peaks were found representing the composite nanofibers’ high crystallinity, probably due to some molecular interactions of PCL and GT that altered the phase composition. Nonetheless, PCL and GT were present in the composite nanofibers.

The most important criterion for creating scaffolds in tissue engineering is identifying their biocompatibility with cells and whether they would provide biochemical or structural cues for cell growth. Previous literature has strongly indicated that cellular attachment, morphology, proliferation, and differentiation were influenced by the scaffold topographic features, such as fiber alignment [21,22]. WJ-MSCs, when cultured on PCL/GT nanofibers, were aligned as opposed to the random orientation seen in the WJ-MSCs cultured on a plate. Besides, the cells quickly integrated with the nanofibers while maintaining their spindle morphology. On the other hand, the resazurin assay in this study demonstrated the PCL/GT nanofiber biocompatibility with the WJ-MSCs, postulating that the nanofibers are safe and not cytotoxic to the cells.

The phenotypic expression of tenocyte-related gene expression was quantitatively determined via PCR. Decorin is a matrix proteoglycan that affects matrix assembly and collagen fiber diameter [44]. Tenascin-C is highly expressed in myotendinous junctions and an early marker in the embryonic tendon [45]. Tenomodulin is predominantly expressed in tenocytes for tendon maturation, while scleraxis is a progenitor cell marker for tendon tissue formation [46,47]. It is thought that aligned nanofibers could affect cell differentiation. Our study found that PCL/GT nanofibers did not exhibit a significant tenogenic effect on WJ-MSCs. Nevertheless, the PCL/GT nanofibers supported tenogenic induction of WJ-MSCs with BMP-12.

Although this study has demonstrated PCL/GT nanofiber potential for tendon/ligament tissue engineering, further studies are still warranted. The current research has not shown the mechanical strength of PCL/GT nanofibers. Another limitation is that nanofibers alone are too weak for tendon/ligament tissue engineering and require an external supporting matrix, such as amniotic membrane, to attain the desired mechanical properties and this is a subject of our future investigation [48]. Furthermore, rigorous nanofibers’ optimization to improve cellular infiltration also presents a great challenge to researchers due to the compact network of nanofibers formed by electrospinning [49].

## 5. Conclusions

This study demonstrated the physicochemical properties and biocompatibility of composite PCL/GT nanofibers for tendon/ligament tissue engineering. Electrospinning has successfully produced aligned PCL/GT nanofibers supporting cellular integration and proliferation. The hydrophobicity of PCL is compensated for by the blending of GT to improve cellular attachment. Moreover, the PCL/GT nanofibers are non-cytotoxic, facilitate alignment and support tenogenic differentiation of WJ-MSCs.

## Figures and Tables

**Table 1:** Elemental analyses of PCL/GT nanofibers by energy dispersive X-ray spectroscopy (EDX). Two-way ANOVA with post-hoc Tukey demonstrated a significant drop of carbon percentage in PCL/GT 0:100 (pure GT) in comparison to the rest of the groups. * *p*-value (<0.05) compared to all PCL/GT nanofibers except PCL/GT 0:100, ‡ *p*-value (<0.05) compared to PCL/GT 100:0 and 70:30.

PCL/GT	100:0	70:30	50:50	30:70	0:100
**Percentage (%), Mean (SD)**					
Carbon	85.9 (7.4)	88.1 (6.4)	82.5 (5.5)	83.5 (8.2)	71.3 (6.8) *
Nitrogen	2.9 (4.0)	5.0 (4.3)	9.3 (5.9)	10.4 (3.8)	15.1 (7.3) ‡
Oxygen	11.3 (7.0)	6.2 (3.3)	8.2 (3.4)	7.8 (4.3)	13.5 (2.9)

## Data Availability

Data sharing not applicable.

## References

[1] GBD 2016 Disease and Injury Incidence and Prevalence Collaborators (2017). Global, regional, and national incidence, prevalence, and years lived with disability for 328 diseases and injuries for 195 countries, 1990–2016: A systematic analysis for the Global Burden of Disease Study 2016. Lancet.

[2] Yang G., Rothrauff B.B., Tuan R.S. (2013). Tendon and ligament regeneration and repair: Clinical relevance and developmental paradigm. Birth Defects Res. C Embryo Today.

[3] Chainani A., Hippensteel K.J., Kishan A., Garrigues N.W., Ruch D.S., Guilak F., Little D. (2013). Multilayered electrospun scaffolds for tendon tissue engineering. Tissue Eng. Part A.

[4] Docheva D., Müller S.A., Majewski M., Evans C.H. (2015). Biologics for tendon repair. Adv. Drug Deliv. Rev..

[5] Dhammi I.K., Haq R.-U., Kumar S. (2015). Graft choices for anterior cruciate ligament reconstruction. Indian J. Orthop..

[6] Santos M.L., Rodrigues M.T., Domingues R.M., Reis R.L., Gomes M.E., Oliveira J., Reis R. (2017). Biomaterials as tendon and ligament substitutes: Current developments. Regenerative Strategies for the Treatment of Knee Joint Disabilities.

[7] Bi F., Shi Z., Liu A., Guo P., Yan S. (2015). Anterior cruciate ligament reconstruction in a rabbit model using silk-collagen scaffold and comparison with autograft. PLoS ONE.

[8] Lim W.L., Liau L.L., Ng M.H., Chowdhury S.R., Law J.X. (2019). Current Progress in Tendon and Ligament Tissue Engineering. Tissue Eng. Regen. Med..

[9] Petrigliano F.A., Arom G.A., Nazemi A.N., Yeranosian M.G., Wu B.M., McAllister D.R. (2015). In vivo evaluation of electrospun polycaprolactone graft for anterior cruciate ligament engineering. Tissue Eng. Part A.

[10] Mohamed R.M., Yusoh K. (2015). A Review on the Recent Research of Polycaprolactone (PCL). Adv. Mater. Res..

[11] Mondal D., Griffith M., Venkatraman S.S. (2016). Polycaprolactone-based biomaterials for tissue engineering and drug delivery: Current scenario and challenges. Int. J. Polym. Mater..

[12] Eshraghi S., Das S. (2010). Mechanical and microstructural properties of polycaprolactone scaffolds with one-dimensional, two-dimensional, and three-dimensional orthogonally oriented porous architectures produced by selective laser sintering. Acta Biomater..

[13] Azimi B., Nourpanah P., Rabiee M., Arbab S. (2014). Poly (lactide-co-glycolide) Fiber: An Overview. J. Eng. Fibers Fabr..

[14] Ciardelli G., Chiono V., Vozzi G., Pracella M., Ahluwalia A., Barbani N., Cristallini C., Giusti P. (2005). Blends of poly-(epsilon-caprolactone) and polysaccharides in tissue engineering applications. Biomacromolecules.

[15] Srinivasa Reddy C., Reddy Venugopal J., Ramakrishna S., Zussman E. (2014). Polycaprolactone/oligomer compound scaffolds for cardiac tissue engineering. J. Biomed. Mater. Res. A.

[16] Scaffaro R., Lopresti F., Maio A., Botta L., Rigogliuso S., Ghersi G. (2017). Electrospun PCL/GO-g-PEG structures: Processing-morphology-properties relationships. Compos. Part A Appl. Sci. Manuf..

[17] Tiwari A.P., Joshi M.K., Kim J.I., Unnithan A.R., Lee J., Park C.H., Kim C.S. (2016). Bimodal fibrous structures for tissue engineering: Fabrication, characterization and in vitro biocompatibility. J. Colloid Interface Sci..

[18] Tiwari A.P., Joshi M.K., Lee J., Maharjan B., Ko S.W., Park C.H., Kim C.S. (2017). Heterogeneous electrospun polycaprolactone/polyethylene glycol membranes with improved wettability, biocompatibility, and mineralization. Colloids Surf. A Phys. Eng. Asp..

[19] Xu Y., Wu J., Wang H., Li H., Di N., Song L., Li S., Li D., Xiang Y., Liu W. (2013). Fabrication of Electrospun Poly(L-Lactide-co-ε-Caprolactone)/Collagen Nanoyarn Network as a Novel, Three-Dimensional, Macroporous, Aligned Scaffold for Tendon Tissue Engineering. Tissue Eng. Part C Methods.

[20] Gorgieva S., Kokol V., Pignatello R. (2011). Collagen-vs. Gelatine-Based Biomaterials and Their Biocompatibility: Review and Perspectives. Biomaterials Applications for Nanomedicine.

[21] Aviss K.J., Gough J.E., Downes S. (2010). Aligned electrospun polymer fibers for skeletal muscle regeneration. Eur. Cell Mater..

[22] Kim H.H., Kim M.J., Ryu S.J., Ki C.S., Park Y.H. (2016). Effect of fiber diameter on surface morphology, mechanical property, and cell behavior of electrospun poly (ε-caprolactone) mat. Fibers Polym..

[23] Law J.X., Liau L.L., Saim A., Yang Y., Idrus R. (2017). Electrospun collagen nanofibers and their applications in skin tissue engineering. Tissue Eng. Regen. Med..

[24] Hotaling N.A., Bharti K., Kriel H., Simon C.G. (2015). DiameterJ: A validated open source nanofiber diameter measurement tool. Biomaterials.

[25] Lamour G., Hamraoui A., Buvailo A., Xing Y., Keuleyan S., Prakash V., Eftekhari-Bafrooei A., Borguet E. (2010). Contact angle measurements using a simplified experimental setup. J. Chem. Educ..

[26] Lim J., Razi Z.R., Law J., Nawi A.M., Idrus R.B., Ng M.H. (2016). MSCs can be differentially isolated from maternal, middle and fetal segments of the human umbilical cord. Cytotherapy.

[27] Lim J., Razi Z., Law J.X., Nawi A.M., Idrus R., Chin T.G., Mustangin M., Ng M.H. (2018). Mesenchymal stromal cells from the maternal segment of human umbilical cord is ideal for bone regeneration in allogenic setting. Tissue Eng. Regen. Med..

[28] Scaffaro R., Lopresti F., Botta L., Rigogliuso S., Ghersi G. (2016). Melt processed PCL/PEG scaffold with discrete pore size gradient for selective cellular infiltration. Macromol. Mater. Eng..

[29] Catania V., Lopresti F., Cappello S., Scaffaro R., Quatrini P. (2020). Innovative, ecofriendly biosorbent-biodegrading biofilms for bioremediation of oil-contaminated water. New Biotechnol..

[30] Beachley V., Wen X. (2009). Effect of electrospinning parameters on the nanofiber diameter and length. Mater. Sci. Eng. C Mater. Biol. Appl..

[31] Kannus P. (2000). Structure of the tendon connective tissue. Scand. J. Med. Sci. Sports.

[32] Parry D.A.D., Craig A.S. (1978). Collagen fibrils and elastic fibers in rat-tail tendon: An electron microscopic investigation. Biopolymers.

[33] Huang Z.M., Zhang Y.Z., Ramakrishna S., Lim C.T. (2004). Electrospinning and mechanical characterization of gelatin nanofibers. Polymer.

[34] Oraby M.A., Waley A.I., El-Dewany E.A., Saad B.M., Abd E.-H. (2013). Electrospun gelatin nanofibers: Effect of gelatin concentration on morphology and fiber diameters. J. Appl. Sci. Res..

[35] Accardi M.A., McCullen S.D., Callanan A., Chung S., Cann P.M., Stevens M.M., Dini D. (2013). Effects of fiber orientation on the frictional properties and damage of regenerative articular cartilage surfaces. Tissue Eng. Part A.

[36] Fernández J., Etxeberria A., Sarasua J.R. (2015). In vitro degradation studies and mechanical behavior of poly (ε-caprolactone-co-δ-valerolactone) and poly (ε-caprolactone-co-L-lactide) with random and semi-alternating chain microstructures. Eur. Polym. J..

[37] Fernández J., Etxeberria A., Ugartemendia J.M., Petisco S., Sarasua J.R. (2012). Effects of chain microstructures on mechanical behavior and aging of a poly (L-lactide-co-ε-caprolactone) biomedical thermoplastic-elastomer. J. Mech. Behav. Biomed. Mater..

[38] Bölgen N., Menceloğlu Y.Z., Acatay K., Vargel I., Pişkin E. (2005). In vitro and in vivo degradation of non-woven materials made of poly (ε-caprolactone) nanofibers prepared by electrospinning under different conditions. J. Biomater. Sci. Polym. Ed..

[39] Chong L.H., Lim M.M., Sultana N. (2015). Fabrication and evaluation of polycaprolactone/gelatin-based electrospun nanofibers with antibacterial properties. J. Nanomater..

[40] Alvarez-Perez M.A., Guarino V., Cirillo V., Ambrosio L. (2010). Influence of gelatin cues in PCL electrospun membranes on nerve outgrowth. Biomacromolecules.

[41] Gautam S., Dinda A.K., Mishra N.C. (2013). Fabrication and characterization of PCL/gelatin composite nanofibrous scaffold for tissue engineering applications by electrospinning method. Mater. Sci. Eng. C.

[42] Lim Y.C., Johnson J., Fei Z., Wu Y., Farson D.F., Lannutti J.J., Choi H.W., Lee L.J. (2011). Micropatterning and characterization of electrospun poly (ε-caprolactone)/gelatin nanofiber tissue scaffolds by femtosecond laser ablation for tissue engineering applications. Biotechnol. Bioeng..

[43] Ghasemi-Mobarakeh L., Prabhakaran M.P., Morshed M., Nasr-Esfahani M.H., Ramakrishna S. (2008). Electrospun poly (ε-caprolactone)/gelatin nanofibrous scaffolds for nerve tissue engineering. Biomaterials.

[44] Chen J.L., Yin Z., Shen W.L., Chen X., Heng B.C., Zou X.H., Ouyang H.W. (2010). Efficacy of hESC-MSCs in knitted silk-collagen scaffold for tendon tissue engineering and their roles. Biomaterials.

[45] Järvinen T.A., Józsa L., Kannus P., Järvinen T.L., Hurme T., Kvist M., Pelto-Huikko M., Kalimo H., Järvinen M. (2003). Mechanical loading regulates the expression of tenascin-C in the myotendinous junction and tendon but does not induce de novo synthesis in the skeletal muscle. J. Cell Sci..

[46] Cserjesi P., Brown D., Ligon K.L., Lyons G.E., Copeland N.G., Gilbert D.J., Jenkins N.A., Olson E.N. (1995). Scleraxis: A basic helix-loop-helix protein that prefigures skeletal formation during mouse embryogenesis. Development.

[47] Shukunami C., Takimoto A., Oro M., Hiraki Y. (2006). Scleraxis positively regulates the expression of tenomodulin, a differentiation marker of tenocytes. Dev. Biol..

[48] Hasmad H., Yusof M.R., Mohd Razi Z.R., Hj Idrus R.B., Chowdhury S.R. (2018). Human amniotic membrane with aligned electrospun fiber as scaffold for aligned tissue regeneration. Tissue Eng. Part C Methods.

[49] Kim S.E., Tiwari A.P. (2020). Three dimensional polycaprolactone/cellulose scaffold containing calcium-based particles: A new platform for bone regeneration. Carbohydr. Polym..

